# Asterics: a simple tool for the ExploRation and Integration of omiCS data

**DOI:** 10.1186/s12859-023-05504-9

**Published:** 2023-10-18

**Authors:** Élise Maigné, Céline Noirot, Julien Henry, Yaa Adu Kesewaah, Ludovic Badin, Sébastien Déjean, Camille Guilmineau, Arielle Krebs, Fanny Mathevet, Audrey Segalini, Laurent Thomassin, David Colongo, Christine Gaspin, Laurence Liaubet, Nathalie Vialaneix

**Affiliations:** 1 Université de Toulouse, INRAE, UR MIAT, 31326 Castanet-Tolosan, France; 2 Université Fédérale de Toulouse, INRAE, Bioinfomics, Genotoul Bioinformatics Facility, 31326 Castanet-Tolosan, France; 3 Plateforme Biostatistique, Genotoul, Toulouse, France; 4 IMT, UMR 5219, Université de Toulouse, CNRS, UPS, 31062 Toulouse, France; 5 Hyphen-Stat, Toulouse, France; 6 GenPhySE, Université de Toulouse, INRAE, ENVT, 31326 Castanet-Tolosan, France

**Keywords:** Web user interface, Statistical analyses, Data integration, Omics

## Abstract

**Background:**

The rapid development of omics acquisition techniques has induced the production of a large volume of heterogeneous and multi-level omics datasets, which require specific and sometimes complex analyses to obtain relevant biological information. Here, we present ASTERICS (version 2.5), a publicly available web interface for the analyses of omics datasets.

**Results:**

ASTERICS is designed to make both standard and complex exploratory and integration analysis workflows easily available to biologists and to provide high quality interactive plots. Special care has been taken to provide a comprehensive documentation of the implemented analyses and to guide users toward sound analysis choices regarding some specific omics data. Data and analyses are organized in a comprehensive graphical workflow within ASTERICS workspace to facilitate the understanding of successive data editions and analyses leading to a given result.

**Conclusion:**

ASTERICS provides an easy to use platform for omics data exploration and integration. The modular organization of its open source code makes it easy to incorporate new workflows and analyses by external contributors. ASTERICS is available at https://asterics.miat.inrae.fr and can also be deployed using provided docker images.

## Background

The rapid development of omics acquisition techniques has induced the production of a large volume of heterogeneous and multi-level omics datasets measured on the same individuals. Information of biological interest is obtained using predictive and exploratory statistical methods performed on one omic level or, more widely from the so-called *integration methods*. The latter methods, which have been increasingly developed in the past few years, aim at providing an understandable representation of the combined information provided by several omic levels or at extracting information about inter-level crosstalk. Some of these methods are already available under the form of packaged software or libraries, and especially as R packages (like **STATegRa **https://doi.org/doi:10.18129/B9.bioc.STATegRa, **mixOmics** [1], **MOFA** [2, 3], **mixKernel** [4, 5], among others; see https://github.com/mikelove/awesome-multi-omics for a more complete list or [6–8]). However, the use of these packages still requires to learn a programming language and to have access to sufficient statistical knowledge to choose method parameters and interpret outputs. Hence, several web based applications have been deployed to perform (sometimes advanced) analyses on omics data, like SHAMAN https://shaman.pasteur.fr/ [9], dedicated to differential analysis of metagenomic data, or iDEP http://ge-lab.org/idep/ [10], GENAVi https://junkdnalab.shinyapps.io/GENAVi/ [11], IDEAmex http://www.uusmb.unam.mx/ideamex/ [12], DIANE https://diane.bpmp.inrae.fr [13], all providing complete analysis pipelines for RNAseq or other count data, mostly oriented toward differential analysis.

We present ASTERICS, an intuitive and interactive web application that aims at making exploratory and integration analysis workflows easily available to biologists and non-statisticians. Contrary to existing tools, ASTERICS

- is not dedicated to a specific type of omics but can perform specialized normalization or differential analyses for different types of omics (including omics obtained as compositional data such as proteomics or metabolomics, bulk transcriptomics as microarray or RNA-seq data, metagenomics,...);
- is not dedicated to a specific type of analysis (e.g., differential analysis) but can handle exploratory analysis (PCA, clustering, etc), differential analysis as well as different types of data edition (including correction of missing values, log-transformation, scaling or sample selection);
- allows the integration of multiple omics, i.e., it includes exploratory analysis able to explain the typology of individuals described by omics and/or characters simultaneously obtained at different levels of the living organisms;
- does not only include a single and straight analysis workflow but allows the user to perform complex analyses workflows with various editions, selections based on statistical analysis results, data normalizations,... while keeping a comprehensive track of the history and dependency of performed operations and analyses.

## Implementation

### Code organization

ASTERICS frontend is developed in Vue.js https://vuejs.org/ and uses the CSS framework Bulma https://bulma.io/. This allows a flexible and modular organization of the different screens and analyses, as well as an easy management of persistant sessions and multiple user sessions.

The backend is controlled with flask https://flask.palletsprojects.com/, pyRserve https://pypi.org/project/pyRserve/ and Rserve https://www.rforge.net/Rserve/ to launch analyses performed with R scripts. R package versions are controlled using **renv** https://CRAN.R-project.org/package=renv to ensure stability and reproducibility of the analyses. An R wrapper runs the function corresponding to the analysis chosen by the user, converts the interactive output plots and the output tables in JSON to serve the interface and maintain a database of datasets and analyses.

The main R packages used for the analyses are: **missMDA** [14], **FactoMineR** [15], **fastcluster** [16], **SOMbrero** [17], **mixOmics**, **VIM** [18], **edgeR** [19, 20], and **sva** [21]. **ggplot2** [22] and **plotly** [23] are also used to produce high quality and interactive graphics.

### Availability and deployment

ASTERICS is distributed under GNU General Public License Version 3 and its source code are available at https://forgemia.inra.fr/asterics/asterics, including recipes for building self-contained docker images. In addition, an open issue repository is available for users wanting to report bugs or suggest improvements https://forgemia.inra.fr/asterics/asterics-issues.

ASTERICS is available online at https://asterics.miat.inrae.fr and does not require any user account creation. It can also be deployed for local usage or on servers using the 3 docker images provided with the project repository https://forgemia.inra.fr/asterics/asterics/container_registry^1^, following the installation instructions provided in the README file of the project repository. In particular, the section “Run docker locally” of this page provides a 5-minute local installation procedure for users having docker installed on their computer.

### Features

**Fig. 1:**
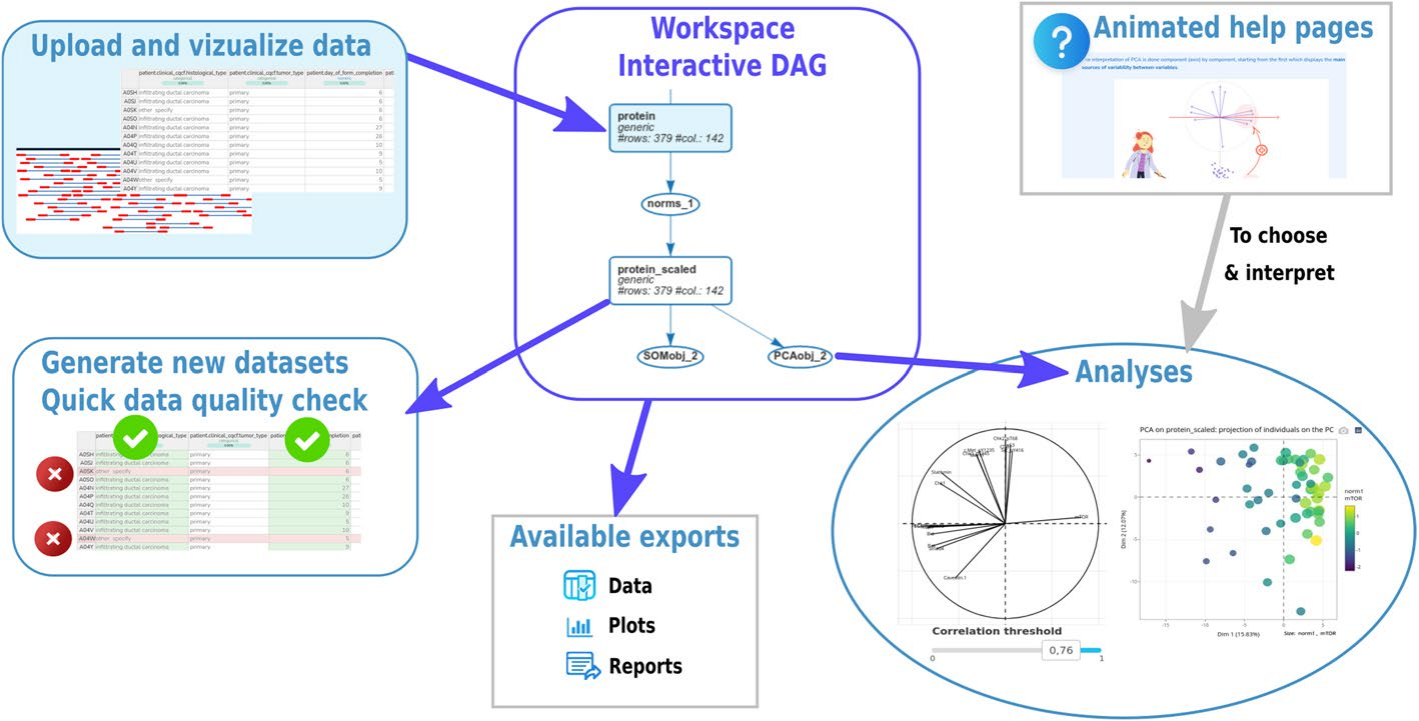
Overview of ASTERICS web interface. The figure illustrates the different features of ASTERICS, including data upload and edition, analyses with interactive plots, comprehensive display of the workspace, exportation of data, plots and reports, and help pages providing advice for choice of analyses and options and tips for interpretation

Figure 1 gives a complete overview of the application: users connect to ASTERICS simply providing an email on which they receive an information about their project ID (and URL), which can be shared with collaborators or re-used later to continue their analysis. The email is also used to warn the user about his/her workspace automatic deletion a few days before it is performed. Workspaces, including data, user email and performed analyses, are deleted after 30 days of inactivity.

Upon connection, users can also automatically load an example dataset extracted from The Cancer Genome Atlas (TCGA, https://www.cancer.gov/tcga) and related to breast cancer as already studied in [24]. In addition, other data derived from this initial example are available at https://doi.org/10.15454/YNMQUY, including information on cancer type and datasets with missing values obtained from the complete dataset with different missing value mechanisms. The complete description of the dataset extractions is also provided in this repository.

Analyses available in ASTERICS are organized into three main menus:

1. **Edit** contains three submenus to
  - perform simple **dataset manipulations** (e.g., selecting rows or columns based on manual selection or on a condition, changing a variable type, etc.);
  - explore, clean or impute **missing values**. Missing values can be either imputed (single PCA imputation as implemented in **missMDA** or *k*-NN imputation as implemented in **VIM**) or rows/columns containing more missing values than a user chosen threshold can be removed from the dataset;
  - perform different types of **normalization** (from a basic scaling to unit variance to more sophisticated normalizations like the TMM and TMMwsp normalizations of **edgeR** for RNA-seq data or the correction of an explicit batch effect as implemented in the “ComBat” method of **sva** [25]);
2. **Explore** contains five submenus to perform simple exploratory analyses of the datasets, including:
  - basic **descriptive statistics** and tests (numerical and graphical summaries for one, two or all the variables, complex plots including up to five variables or correlation plot between all variables);
  - **PCA** (with plots for individuals that can be interactively combined with additional metadata variables and plots for variables that can be filtered based on a given correlation threshold) and its extension to mixed data [26];
  - **heatmap** or dotplot (for which rows and/or columns can be organized by a hierarchical clustering and that can also be combined with metadata describing individuals);
  - **clustering** (based on a fast version of hierarchical clustering [16] or on *k*-means). Outputs of this analysis include a heatmap and a PCA individual plot based on the initial data where the clusters are displayed with colors; the PCA plot can also be interactively combined with external information to compare the clustering with known groups of individuals;
  - **self-organizing maps** [27], a method combining clustering and dimension reduction, that offers various graphics usable to obtain a summarized representation of the typology of the individuals in a dataset;
3. **Integrate** contains all analyses that are based on data coming from at least two different datasets, in a generic sense (these datasets can be omics datasets or other types of data, including metadata). This includes both analyses designed to explore relationships between at least two datasets containing, each, multiple variables (PLS and MFA) or analyses designed to compare all numeric variables in a given dataset with respect to the levels of a categorical variable from another dataset (PLS-DA and differential analysis). **Integrate** contains 4 submenus to perform data integration with
  - **PLS** [28], a method unraveling relationships between two numeric datasets acquired on the same matched individuals. Similarly to PCA, it creates composite variables (e.g., linear combinations of the original variables) in each dataset, so as to maximize the covariance between these composite variables;
  - **PLS-DA** [29], a method, based on PLS, integrating a numeric dataset with a categorical variable. The categorical variable is used as an information on classes (or conditions) on individuals and the method creates composite variables from the numeric dataset so that they separate at best the individuals according to their classes. As for differential analysis, this helps highlight the variables the most involved in the difference between conditions;
  - **MFA** [30], a generalization of the PCA for cases where individuals are described by several groups of variables, including categorical ones. The method weights each group of variables in a data-driven fashion so as to balance the contribution of each group in the global PCA;
  - **differential analysis** of all the numeric variables in a given dataset with respect to the level of an external categorical variable. In this analysis, statistical tests are performed on all the variables of a numeric dataset to compare their means with respect to the levels of a categorical variable (thus describing conditions on individuals). The differential analysis is automatically adjusted to the type of data provided by the user: for generic or continuous data, a normality test can be performed to choose between 1-way ANOVA or Kruskal-Wallis test. For count data, the test defaults to negative binomial GLM tests as implemented in **edgeR** [20]. Also, posthoc tests, comparing all pairs of the factor levels can be performed when the global test is found positive.
  Note that PLS-DA and differential analysis have the same purpose but using two very different types of methods: PLS-DA is an exploratory approach using all the numeric variables in a multivariate fashion while differential analysis performs a test for every numeric variable independently and provides statistical guarantee (i.e., provides a *p*-value to control the probability of false rejection) on the result. The first three (factorial) analyses provides outputs similar to PCA (individual and variable plots, all interactive and that can be customized with external information), in addition to some other plots specific of each analysis (for instance, the individual plot of the MFA can be complemented with an information about the contribution of each type of data on the individual positions).

In addition, a menu **My workspace** summarizes the current state of the datasets, analyses and dependencies between them by an interactive directed acyclic graph (DAG). It also includes:

- a list of all datasets currently available, which includes the possibility to remove some, along with all the analyses that have been performed on them, to export a selection (in CSV or RDA), or to add new ones. During importation, the user can specify if the dataset corresponds to a specific type of omic (e.g., “Sequencing data (RNA-seq, miRNA,...) counts” or “metagenomics composition (compositional data)”) and if these data have been previously normalized or log-transformed. These choices automatically drive sound options in the edition, normalization, or differential analysis menus, with understandable warnings or errors if the user does not perform the best analysis corresponding to what he/she has declared during importation (e.g., for RNA-seq data, users are advised to perform a TMM normalization before performing differential analyses and differential analysis is automatically performed with edgeR GLM model unless the counts have been log-transformed),
- a list of all already performed analyses, together with their parameters. Analysis can also be exported in RDA format or as HTML reports. The latter include the workflow DAG, all numeric and graphical results obtained in the analysis and an information on the version of ASTERICS that has produced these results.

ASTERICS has been designed to help non-statisticians and beginners to choose the analyses and options the most appropriate to his/her question. By clicking on one of the three main menus, all analyses available in this menu are described in a simple way with plots and short texts to help the user understand the purpose of the analysis. In the different screens, a question mark button  points to a documentation explaining how to set options and how to interpret results of the different screens, with short texts, understandable plots or short videos. This documentation is also gathered in a single document available at https://asterics.pages.mia.inra.fr/user_documentation/ that provides descriptions of the functions and methods used in the backend to perform the analyses, along with the way the options have been set for these functions. The user documentation includes two case studies, one using the example datasets available in the interface^2^ and the other using an in-house dataset obtained to study piglet survival [31–33].^3^

## Results

This section presents a case study derived from the case study “Piglet” included in ASTERICS user documentation. This study is focused on fetal development in late gestation in pig species and addresses the question of delayed development in mammalian that may impact survival ability at birth. Two ages at late gestation were studied, 90 and 110 days (birth is expected at 114 days). Two genetic lines were included, LW for Large White, with higher risk of delayed development, and MS for Meishan, more robust. Studied piglets were of four genotypes: LWLW, MSMS, for pure breeds (mother and father are of the same genotypes), and the crossed piglets LWMS and MSLW (father then mother genotype). Here, we perform an analysis of three metabolomics datasets, respectively obtained on plasma, urine, and amniotic fluid samples of 444 piglets, which is an extension of the study of [33].

The workspace of the case study is made available under UID piglet_usecase on ASTERICS.^4^ Datasets are described in [33] and correspond to a metabolomic analysis performed with $^1$H NMR technique. Raw data ($^1$H NMR spectra) are available in the metabolights database [34] under accession number MTBLS1541. Spectra were processed with the **ASICS** package to identify and quantify metabolites as described in [33] and metabolite quantifications together with a metadata dataset containing information on the design of the experiment have been made available at https://doi.org/10.57745/TCKSTD.

### Dataset importation and correction

**Fig. 2:**
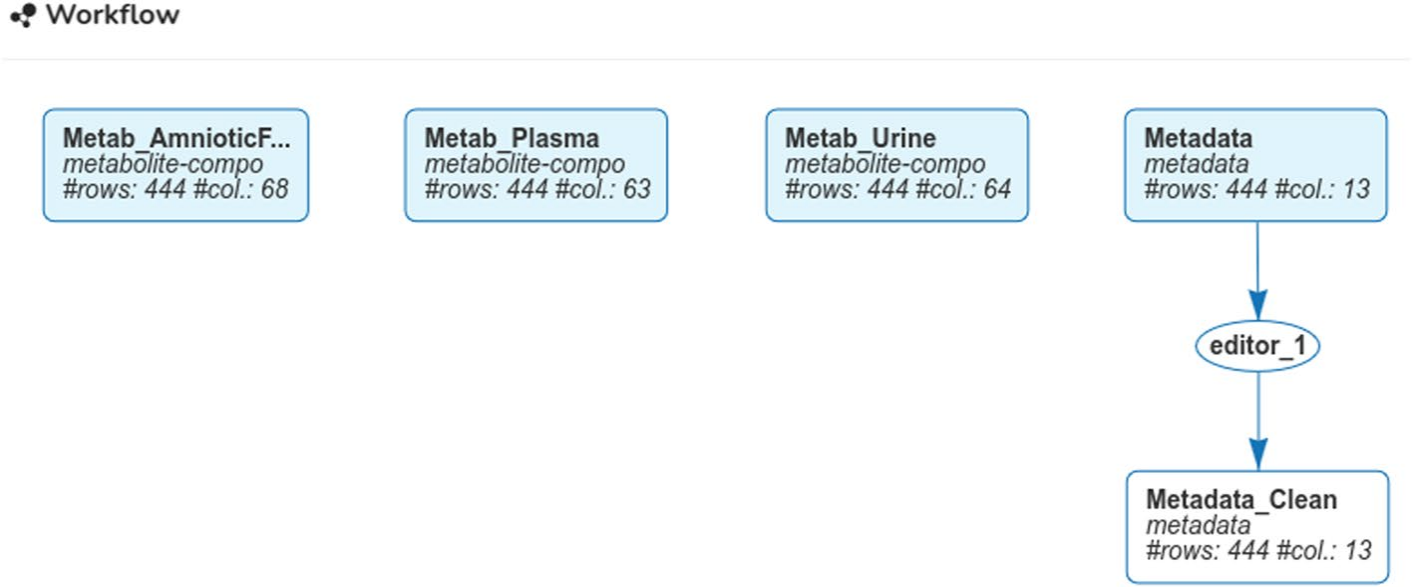
Workflow from the **My workspace** menu, as obtained after the importation of the four CSV files and the change of a variable type in the last dataset “Metadata” (that contains the uploaded data describing the design of the experiment)

From **My workspace**, the four datasets (three metabolite quantifications and information on the experimental design) are imported from CSV files using the button “Add” in the panel “All datasets”. For metabolite quantifications, the original datasets were transposed during the upload to stick to ASTERICS requirements that the individuals are in rows. In the experimental design file, one of the variable (N_mother, reporting the mother identifier) was encoded as numerical in the CSV file. The menu “Edit/Dataset edition” allows to correct that problem, using the option “Change variable (column) types”. These operations resulted in the workflow DAG of Fig. 2, as obtained from the menu **My workspace**. The four imported datasets are displayed in blue in rectangular nodes, whereas the corrected design of the experiment is displayed in white, also in a rectangular node. The edition process is also displayed in white, with a round node, as for all the analyses. This simple representation allows to easily understand the relationships between datasets and analyses.

### Exploratory analysis of the design of the experiment

First, simple exploratory analyses were first performed to understand the structure of the metadata file.

Using the menu “Explore/Explore variables in a dataset/All variables in a dataset”, the content of the design of the experiment dataset (named Metadata_Clean) was summarized. From **My workspace**, the results can be obtained by simply clicking of the node corresponding to the analysis “UnivariateDataset_1” and further on the button “More” in the panel that opens on this action. For numerical variables, statistical summaries (min, quartiles, mean, maximum, standard deviation, number of missing values, number of unique values) are displayed in a table and the distributions are represented by boxplots (that can be scaled or not, to account for possible differences in scales between the numeric variables). Normality tests (Shapiro-Wilk) indicated that all variables significantly deviate from the normal distribution. For categorical variables (factors), statistical summaries (number of missing values, number of unique values, percentage and number of individuals in the mode level) are displayed in a table and the distributions are visualized with colored barplots. This simple analysis showed that the distributions of age, sex and genotype of the mother were balanced in the experiment, whereas the distribution of the father genotype was unbalanced (383 LW versus 61 MS). This issue is due to the fact that sows were inseminated with mixed LW+MS semen but LW semen shows a higher efficiency for reproduction. On the contrary, the mother genotype was fully controlled by the experimental design.

### Relation between 3-fluid metabolome and stage of gestation

The last part of the analysis consisted in the analysis of the stage of gestation effect on the metabolome. Contrary to what was performed in [33] to address this question (PLS-DA analyses performed independently on each of the fluid), we performed an integrated analysis combining the three fluids using MFA. The effect of the stage of gestation (“Age”) was further investigated on plasma metabolome using a differential analysis.

**Fig. 3:**
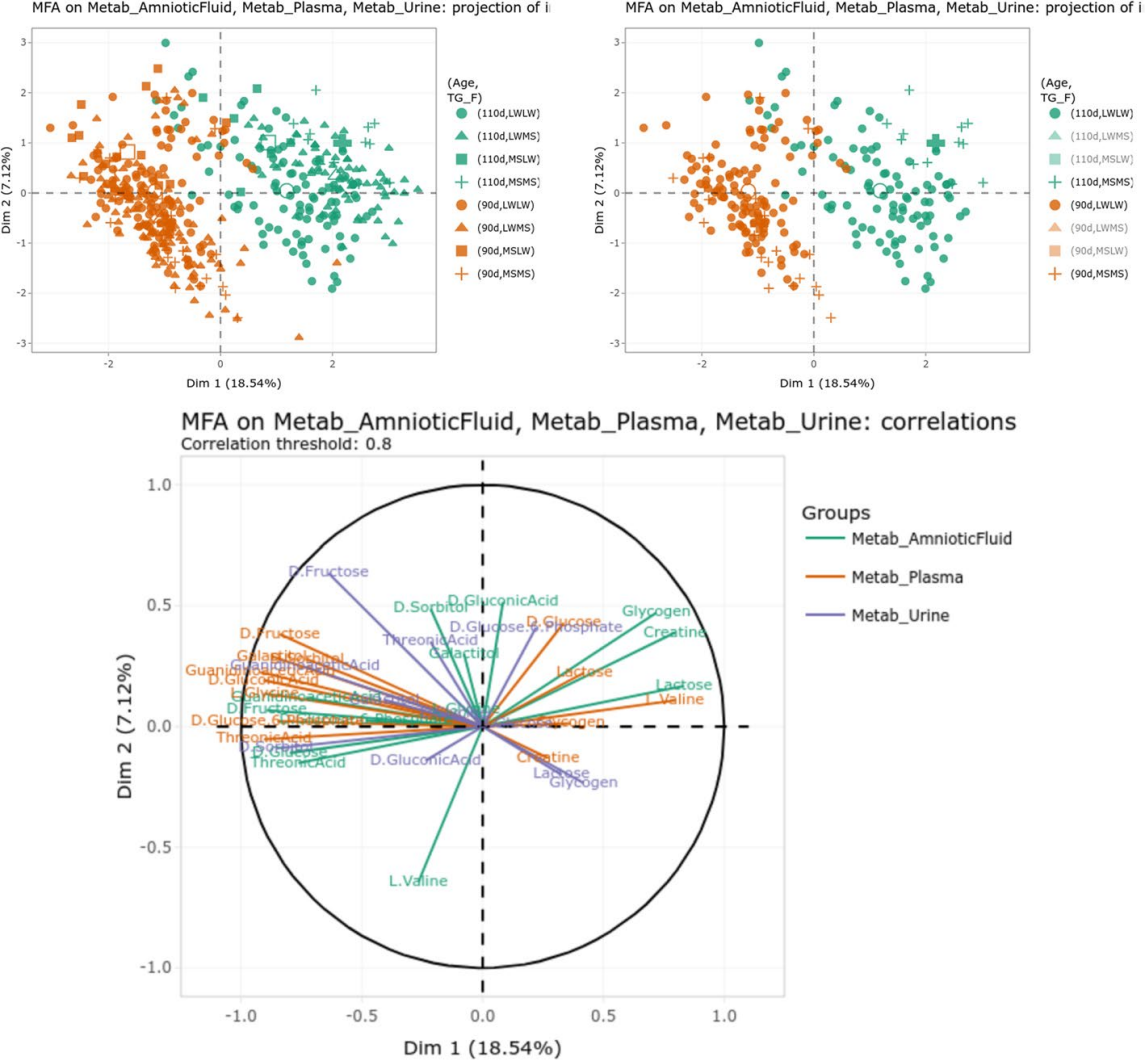
Individual (top) and variable (bottom) plots obtained from the MFA of the three metabolomics datasets. The right individual plot is interactively obtained from the left one by clicking on the mixed genotype icons of the legend to remove the corresponding data from the plot and increase readability

The menu “Integrate/Integrate dataset with MFA” allowed us to perform a MFA for the three metabolomics datasets. The individual plot (first two axes of the MFA with variables “Age” and “TG_F” superimposed as colors and shapes respectively, Fig. 3, top) showed a good discrimination of the stage of gestation on the first axis but not of the fetus genotype “TG_F” (even interactively removing the mixed genotypes from the plot as shown on the right). However, combining the day of gestation (for color) with the mother genotype (“TG_M”) allowed us to show a separation between the two mother genotypes at 110 days of gestation on the first axis: Meishan sows (MS) were positionned further on the right of this axis, confirming its relation with piglet maturity (The Meishan breed is expected to be robust and more mature at birth).

The individual plot was put in relation with the variable plot (first two axes of the MFA, correlation threshold of 80%, Fig. 3, bottom), which allows to easily identify metabolites up-concentrated at 90 or 110 days for the different fluids. In particular, glycose-6-phosphate and lactose are up-concentrated in the three fluids at 110 days of gestation whereas fructose and guanidinoacetate (guanidinoacetic acid) are down-concentrated in late (110 days of) gestation. This confirms previous finding of [33], which also found these metabolites evolving in the reported directions using independent mixed models for the three fluids. Interestingly, lactose was not found differentially expressed by mixed model in urine in [33], whereas, the MFA shows a clear (even if weaker) relation of this metabolite with the first axis. Also notable, valine and D-glucose showed opposite evolution in amniotic fluid and plasma (they are less concentrated in amniotic fluid and more concentrated in plasma at 110 days of gestation). In addition, D-glucose is also up-concentrated at 110 days in urine (but to a lesser extent than plasma). This, combined with the expected transfer between the three fluids (from plasma to urine for excretion and to amniotic fluid as a source of nutrients for the fetus), seems to indicate that these metabolites tend to be used by the fetus in late gestation: glucose is used for energy storage (under the form of glycogen [35]) and essential amino acids, like valine, have already been reported to decrease in amniotic fluid during late gestation in Humans [36].

**Fig. 4:**
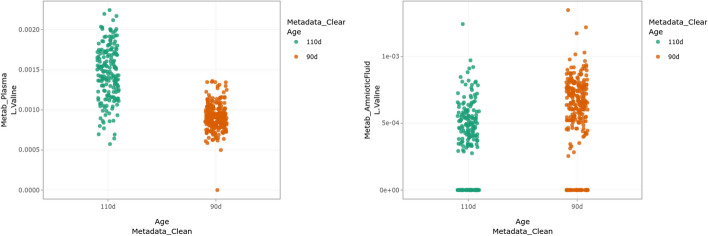
Concentration of valine in plasma (left) and amniotic fluid (right) at the two ages of gestation

The menu “Explore/Explore variable in a dataset/2 variables” can be used to further visualize the impact of the stage of gestation on metabolites found important to drive the first axis of the MFA. For instance, used on valine in plasma and amniotic fluid, this menu generates the graphics in Fig. 4, which confirms the findings of the MFA and shows, in addition, that valine is much more concentrated in plasma than in amniotic fluid. Since valine was also not detected in urine, these results tend to indicate that valine, which is not excreted, is used by the fetus in late gestation. Note that both plots can be saved using the corresponding icon for further inclusion in exported report (see next section).

Finally, using the menu “Integrate/Differential analysis”, a differential analysis of the plasma metabolome with respect to the day of gestation (“Age”) is performed with nonparametric tests (correction for multiple test performed with the Benjamini-Hochberg procedure [37]). The panel “Extract dataset” in this workflow allowed us to export down- and over-concentrated metabolites found differential for this analysis (FDR $<5$%). These results confirm that (as already discussed before) valine is significantly more concentrated in plasma at 110 days of gestation (adjusted *p*-value = 1.674e-52).

Detailed results can be exported as CSV files from **My workspace** and are made available for the reader in the data repository https://doi.org/10.57745/TCKSTD. Using these results in MetaboAnalyst 5.0 https://www.metaboanalyst.ca/ [38], we also confirmed the Galactose metabolism pathway as the most significant pathway for metabolites down-concentrated at 110 days of gestation. This confirms previous findings by [33]), which also reported that most of the metabolites in this pathway were also found differential in the other two fluids.

### Saving results

Finally, in **My workspace**, the analyses “UnivariateDataset_1”, “bivariateAnalysis” and “MFAobj_1” were selected in the panel “All analyses” and a report was obtained by clicking on the button “Export report”. These reports are also made available in the data repository https://doi.org/10.57745/TCKSTD.

## Discussion and conclusions

ASTERICS makes the analysis and combined analyses of datasets easily accessible to people not expert in statistics, data science and programming. Other tools like Orange [39] might provide a more extensive panel of statistical and machine learning methods but they do not offer the same level of interactivity in plots, do not include data processing methods and analyses specific to certain omics, and require a local installation. Our use case study showed that ASTERICS is able to perform analyses in omics integration settings and to reproduce findings in just a few clicks. Results and plots can be customized, including the dataset and variable names, to produce high-quality outputs ready for publication (CSV files, analysis report in HTML format and figures).

Currently, the version of ASTERICS deployed online is designed to handle small-to-medium datasets (typically of the size of standard RNAseq data) due to limitations in RAM of the deployment server and to the uploading of data over https in uncompressed form. Local deployment does not have this limitation (it is only limited by the computating capacity of the local deployment machine).

Currently, ASTERICS is designed to perform the most common statistical analyses on the most common omics (batch transcriptomics from microarray or RNA-seq experiments, metagenomics, metabolomics or proteomics, in particular as compositional datasets, etc). However, the tool has been programmed in a modular way (both in the frontend and backend) with a full developer documentation so as to allow the extension of ASTERICS to other analyses. Future developments are expected to include more advanced workflows (MOFA, DIABLO, kernel methods), analyses helpful for interpreting results such as gene enrichment analysis and network inference, as well as more recent omics such as single-cell RNA-seq.

## Availability and requirements

*Project name*: ASTERICS

*Project home page*: https://forgemia.inra.fr/asterics/asterics. (also archived on software heritage under SWHID https://archive.softwareheritage.org/swh:1:dir:7fbddc80f287596a6472d29800681ef74b682dfe).

*Operating systems*: platform independent.

*Programming languages*: R, Python, JavaScript, HTML.

*Other requirements*: docker for local deployment and none for web usage.

*License*: GPL 3.

*Any restrictions to use by non-academics*: none.

## Data Availability

The source code of ASTERICS is released under GPL-3 license at https://forgemia.inra.fr/asterics/asterics and docker images for its local deployment are also available in this repository. $^1$H NMR spectra on which the case study is based are available in the metabolights database [34] under accession number MTBLS1541. Processed data (metabolite quantifications), experimental design and output reports of ASTERICS are available at https://doi.org/10.57745/TCKSTD. The case study is fully accessible at https://asterics.miat.inrae.fr/workspace/piglet_usecase.

## Footnotes

1 A fourth docker image is provided to be used as a base image for R and packages but is not useful for deployment.

2 https://asterics.pages.mia.inra.fr/user_documentation/case-study-breast-cancer.html.

3 https://asterics.pages.mia.inra.fr/user_documentation/case-study-piglet.html.

4 Directly accessible at https://asterics.miat.inrae.fr/workspace/piglet_usecase.
